# PKR acts early in infection to suppress Semliki Forest virus production and strongly enhances the type I interferon response

**DOI:** 10.1099/vir.0.007336-0

**Published:** 2009-06

**Authors:** Gerald Barry, Lucy Breakwell, Rennos Fragkoudis, Ghassem Attarzadeh-Yazdi, Julio Rodriguez-Andres, Alain Kohl, John K. Fazakerley

**Affiliations:** The Roslin Institute and Royal School of Veterinary Studies, College of Medicine and Veterinary Medicine, University of Edinburgh, Summerhall, Edinburgh EH9 1QH, UK

## Abstract

The double-stranded RNA-activated protein kinase (PKR) is a key regulator of protein translation, interferon (IFN) expression and cell survival. Upon infection of vertebrate cells in continuous culture, the alphavirus Semliki Forest virus (SFV) initiates apoptosis and IFN synthesis. To determine the effect of PKR on SFV infection, we studied the course of infection in wild-type (wt) mice, mice with a genetic deletion of PKR (PKR^−/−^) and mouse embryo fibroblasts (MEFs) derived from these mice. In MEFs, PKR delayed virus protein synthesis, production of infectious virus and caspase-3-activated cell death and reduced the yield of infectious virus by 90 %. Small interfering RNA suppression of PKR levels in NIH-3T3 cells also reduced virus production and apoptosis. In MEFs, PKR was not required for initiation of IFN-*β* gene transcription, but contributed strongly to the magnitude of this response. Levels of IFN-*β* transcripts in PKR^−/−^ MEFs at 8 h were 80 % lower than those in wt MEFs and levels of functional IFN at 24 h were 95 % lower. Following infection of wt and PKR^−/−^ mice, SFV4 and SFV A7(74) were avirulent. PKR increased levels of serum IFN and the rate of clearance of infectious virus from the brain. In summary, in response to SFV, PKR exerts an early antiviral effect that delays virus protein production and release of infectious virus and, whilst PKR is not required for induction of apoptosis or activation of the type I IFN response, it strongly augments the type I IFN response and contributes to clearance of infectious virus from the mouse brain.

## INTRODUCTION

Alphaviruses (family *Togaviridae*) produce disease in a variety of animals and replicate in invertebrates, birds and mammals. In humans, alphaviruses produce encephalitis or arthralgia. Large epidemics can occur, as witnessed by the current epidemic of chikungunya virus (Powers & Logue, 2007). Alphavirus encephalitis has been studied extensively in Semliki Forest virus (SFV)- and Sindbis virus (SV)-infected mice and these infections serve as general models to study virus encephalitis (Fazakerley, 2004; Griffin, 2005).

Alphaviruses are single-stranded, positive-sense, enveloped RNA viruses with a genome of approximately 11.5 kb with a 5′ cap and a 3′ poly(A) tail. The 5′ two-thirds of the genome encodes four non-structural proteins (nsP1–4). These are translated directly from the full-length 42S genomic RNA and form the virus replicase responsible for RNA replication and transcription (Takkinen, 1986). The virus structural proteins, capsid and envelope glycoproteins E1, E2 and E3 are encoded at the 3′ end of the genome and translated from a subgenomic 26S RNA, which is transcribed by the mature replicase complex using a promoter in the full-length genome cRNA (Lemm & Rice, 1993b; Liljeström & Garoff, 1991). Alphavirus infection results in shutoff of host-cell protein synthesis, which is usually apparent 2–4 h post-infection (Frolov & Schlesinger, 1994a). After this, virus subgenomic 26S RNA continues to be translated and virus structural proteins are the main products of cellular translation. This property has been exploited in the development of alphavirus expression vectors (Liljeström & Garoff, 1991; Xiong *et al.*, 1989). Shutoff of host-cell protein synthesis is associated with phosphorylation of eIF2*α* and formation of stress granules, which sequester mRNA preinitiation complexes (Kedersha *et al.*, 1999; McInerney *et al.*, 2005). These stress granules are disassembled in the vicinity of SFV replication (McInerney *et al.*, 2005). The alphavirus subgenomic message contains a translational enhancer, an RNA secondary structure that allows initiation of translation of virus subgenomic transcripts in the absence of eIF2*α* (Frolov & Schlesinger, 1994b; Sjöberg *et al.*, 1994; Ventoso *et al.*, 2006). Mature virions are released by budding at the plasma membrane or by fusion of intracellular virus-containing vacuoles with the plasma membrane (Pathak *et al.*, 1976).

In vertebrate cells in continuous culture, alphavirus infection generally results in apoptotic cell death between 24 and 48 h post-infection (Glasgow *et al.*, 1997; Scallan *et al.*, 1997). SFV-induced apoptosis does not require virus structural proteins, is p53-independent, is triggered by RNA replication and involves activation of a mitochondrial, caspase-8-dependent, Bak- and Bid-mediated death-signalling pathway (Glasgow *et al.*, 1998; Grandgirard *et al.*, 1998; Urban *et al.*, 2008). Mutations in SFV or SV nsP2 can attenuate this cytopathic effect (Lundstrom *et al.*, 2003; Tamm *et al.*, 2008). Generally, alphaviruses are very sensitive to interferon (IFN) and pre-treatment of cells with IFN prior to infection prevents replication (Deuber & Pavlovic, 2007). In mice, intraperitoneal (ip) inoculation of SFV results in a high-titre plasma viraemia, which triggers an IFN response and seeds virus to the central nervous system (CNS) (Bradish *et al.*, 1975; Bradish & Titmuss, 1981). In the absence of a type I IFN response, SFV spreads rapidly to infect many tissues, and animals die within 48 h (Fragkoudis *et al.*, 2007). Absence of a functional type I IFN response also results in rapid death following infection with other alphaviruses (Grieder & Vogel, 1999; Ryman *et al.*, 2000). The key elements of this IFN-mediated protective response remain to be determined.

Double-stranded (ds) RNA-activated protein kinase (PKR) is an extensively studied cellular protein important in normal cell function, cell stress and cell death (Garcia *et al.*, 2006; Sadler & Williams, 2007). In mammalian cells, PKR is expressed constitutively, but levels vary according to cell type and differentiation (Haines *et al.*, 1993). The best-known activator of PKR is dsRNA generated during RNA virus replication. PKR binds dsRNA via its N-terminal domain. PKR can also be activated by cellular RNAs and the cellular PKR activator protein. PKR is activated in many virus-infected cells, tumour cells and neurons during neurodegenerative disease (Jagus *et al.*, 1999; Peel & Bredesen, 2003). As availability of eIF2*α* is a rate-limiting step in the initiation of translation, PKR is a key regulator of protein synthesis. PKR also activates the IKK complex, leading to nuclear translocation of NF-*κ*B and activation of IFN, pro-inflammatory cytokines and cell death or survival responses (Clemens *et al.*, 1993; Gil *et al.*, 2000; Henry *et al.*, 1994; Kumar *et al.*, 1994; Lee & Esteban, 1994; Lemm & Rice, 1993a; Mundschau & Faller, 1995). Inhibition of translation, production of IFN and cytokines and triggering of apoptosis combine to make PKR a powerful antiviral molecule and several viruses have evolved strategies to antagonize it (Langland *et al.*, 2006).

The role of PKR in virus infections has been studied by using dominant-negative proteins and two lines of genetically modified mice (Abraham *et al.*, 1999; Yang *et al.*, 1995). Alphavirus infections, including SFV, are known to activate PKR, resulting in phosphorylation of eIF2*α* (Ventoso *et al.*, 2006). A number of studies have examined the role of PKR in SV infection (Gorchakov *et al.*, 2004; Ryman *et al.*, 2002, 2005; Sawicki *et al.*, 2003), but there have been no detailed studies of other alphavirus infections. We have shown recently that mice without a functional type I IFN system succumb rapidly to widespread SFV infection (Fragkoudis *et al.*, 2007). To investigate the role of PKR in SFV infection, the course of infection was compared in wild-type (wt) mice, mice with a disruption of the PKR gene (PKR^−/−^) and mouse embryo fibroblasts (MEFs) derived from these mice. In MEFs, PKR did not affect SFV RNA levels, but delayed virus protein translation and production of infectious virus. Whilst not required to initiate apoptosis or IFN responses, PKR extended the life of infected MEFs and strongly augmented IFN responses. In SFV-infected mice, PKR contributed to IFN induction and clearance of infectious virus from the brain, but was not required for protection against overwhelming systemic infection (Fragkoudis *et al.*, 2007).

## METHODS

### Virus.

The full-length SFV4 cDNA was a kind gift from Professor P. Liljeström (Karolinska Institute, Sweden). Infectious virus was generated from the pSP6-SFV4 plasmid (Liljeström & Garoff, 1991). Briefly, the plasmid was linearized with *Spe*I and capped transcripts were produced by *in vitro* transcription (SP6) in the presence of m7GpppG cap analogue. RNA was electroporated into BHK-21 cells using two consecutive 140 V square-wave pulses with a pulse length of 25 ms. Virus stocks were collected after 24 h at 37 °C, titrated and used to infect fresh BHK cells (m.o.i. of 0.1). At 24 h, the working stock was harvested; to remove factors released into the virus culture supernatant, this was purified by passage through a sucrose gradient followed by precipitation and washing (Fazakerley *et al.*, 1993). We have previously described the origin and preparation of the A7(74) strain of SFV (Fazakerley *et al.*, 1993).

### Mice and MEFs.

C57BL/6×129/Sv/Ev wt mice and C57BL/6×129/Sv/Ev mice with a disruption of the PKR gene (PKR^−/−^) (Yang *et al.*, 1995) were a kind gift from Dr Jovan Pavlovic (University of Zürich, Switzerland). All mice were bred in the Centre for Infectious Diseases, University of Edinburgh. The genetic status of all mice was confirmed and monitored by PCR. Mice were kept under specific-pathogen-free and environmentally enriched conditions, with food and water supplied *ad libitum*. All breeding and animal experimentation was agreed by the University of Edinburgh Ethical Review Committee and carried out under the authority of a UK Home Office licence. Mice were inoculated ip with virus and were monitored for clinical disease twice daily for 14 days. To determine brain virus titres, groups of mice were inoculated ip with virus and, under terminal anaesthesia, were perfused with PBS prior to removal of the brain; brain virus titres were determined as described previously (Fazakerley *et al.*, 1993). MEFs were derived from 13.5-day-old embryos and maintained in Dulbecco's modified Eagle's medium containing 100 U penicillin μl^−1^, 100 μg streptomycin ml^−1^ and l-glutamine (2 mM) (DMEM) with the addition of 10 % fetal calf serum (FCS). For infection, MEFs were incubated in 5 % CO_2_ at 35 °C until 80–90 % confluent; growth medium was removed and the cells were inoculated for 1 h at room temperature with virus or control medium. Monolayers were washed, covered with DMEM plus 10 % FCS and incubated at 37 °C for the required time. Replicate experiments were carried out using different primary cultures of MEFs.

### PCR to determine genomic status of PKR mice and MEFs.

The genotypes of wt and PKR^−/−^ mice and MEFs derived from these mice were determined by PCR. In a total volume of 50 μl, reactions contained 1 μl (50 pM) forward primer, 1 μl (50 pM) reverse primer, 1 μl (10 mM) dNTPs, 3 μl (25 mM) MgCl_2_, 5 μl 10× PCR buffer, 36.6 μl distilled H_2_O, 2 μl cDNA and 2.5 U *Taq* DNA polymerase (all reagents from Invitrogen). To reduce variation, a master mix was created without *Taq* polymerase, which was added after a hot start of 94 °C for 3 min. Samples were then cycled through 94 °C for 15 s, 55 °C for 30 s and 72 °C for 2 min for 30–35 cycles, followed by 72 °C for 10 min for the final extension phase. A band at 369 bp on a 1.8 % agarose gel indicated an intact PKR gene. The primers used were 5′-CGAACAAGGAGAACAGGAACT-3′ and 5′-TAAAGGAAAACCACGGCAGA-3′.

### RNA extraction and quantitative (Q) PCR.

RNA was extracted from cell pellets by using a Qiagen RNeasy Mini kit. RNA quality and quantity were assessed by using an Agilent Bioanalyzer. High-quality RNA samples were reverse-transcribed (McKimmie & Fazakerley, 2005). Samples to be compared directly were reverse-transcribed at the same time, using the same master mix (Invitrogen). Test samples and standards were always assayed in triplicate. Briefly, in a total volume of 20 μl (made up in RNase-free water), reaction mixes contained 40 pM primer A, 40 pM primer B, 40 mM dNTPs, 10× buffer, 27 mM MgCl_2_, 1 : 20 000 SYBR Green (Biogene), 5 U FastStart *Taq* (Roche) and 2 μl cDNA. Tubes were heated to 94 °C for 5 min; the PCR was then cycled through 94 °C for 20 s, 62 °C for 20 s and 72 °C for 20 s for 40 cycles on a RotorGene 3000 (Corbett Research). Standard curves using serial dilutions of plasmids containing the target sequences were used to quantify the RNA in unknown samples. Samples were normalized to total RNA determined on an Agilent Bioanalyzer (Brown *et al.*, 2003). Sequences of the primers used in the assay were as follows: IFN-*α* (a primer based on IFN-*α*_4_), 5′-AGGACAGGAAGGATTTTGGA-3′ and 5′-GCTGCTGATGGAGGTCATT-3′; IFN-*β*, 5′-CACAGCCCTCTCCATCAACT-3′ and 5′-GCATCTTCTCCGTCATCTCC-3′; *β*-actin, 5′-CGTTGACATCCGTAAAGACC-3′ and 5′-CTGGAAGGTGGACAGTGAG-3′; SFV nsP3, 5′-GCAAGAGGCAAACGAACAGA-3′ and 5′-GGGAAAAGATGAGCAAACCA-3′.

### Radiolabelling of cellular protein.

Newly synthesized proteins were labelled with [^35^S]methionine and [^35^S]cysteine ([^35^S]Met/Cys; GE Healthcare). PKR^−/−^ and wt MEFs were grown in six-well plates until approximately 90 % confluent. The cells were infected at time 0 and then placed back in the incubator. Thirty minutes before each time point, medium was removed from the wells and 1 ml starvation medium (DMEM without sodium pyruvate, methionine or cysteine; Invitrogen) was added. At each time point, starvation medium was removed and replaced with starvation medium containing 25 μCi (925 kBq) [^35^S]Met/Cys ml^−1^ for 30 min. The medium was then removed and the cells were lysed with Laemmli buffer. Protein concentrations were measured and equal amounts were run on a 12 % polyacrylamide/SDS gel. Protein bands were visualized by exposure to ECL Hyperfilm (GE Healthcare).

### Virus growth curves, cell viability and caspase-3 activation.

PKR^−/−^ and wt MEFs were grown in six-well plates at 37 °C and 5 % CO_2_ until approximately 90 % confluent. Triplicate cultures were infected with SFV4 and, at each time point, a sample of medium was collected and replaced with fresh medium. The virus titre was determined by plaque assay (Fazakerley *et al.*, 1993). Cell viability was measured by using Cell Proliferation Reagent WST-1 (Roche), and caspase-3 activation by using an enzyme-based reporter assay (Sigma). Levels of PKR were reduced in NIH-3T3 cells by transfection (Lipofectamine 2000; Invitrogen) of 100 nM small interfering RNA (siRNA) directed against PKR (sc-36264; Santa Cruz); 100 nM *Silencer* siRNA (AM4611; Ambion) was used as a control.

### Western blotting.

Cells were lysed [50 mM Tris, 150 mM NaCl, 1 % NP-40, 0.25 % SDS, 1 mM dithiothreitol (pH 8), containing 1/100 Protease Inhibitor Cocktail (Sigma)], homogenized, clarified by centrifugation at 10 000 ***g*** for 2 min and denatured at 100 °C (5 min). Lysates were run on a 10 % polyacrylamide/SDS gel and blotted onto nitrocellulose. Proteins were detected by using a rabbit polyclonal anti-PKR antibody (Santa Cruz) and a rabbit anti-actin antibody (Sigma), followed by a goat anti-rabbit antibody conjugated to horseradish peroxidase (Cell Signaling Technology); bands were visualized by using a SuperSignal West Pico Chemiluminescence kit (Perbio).

### IFN assays.

Infectious virus was first inactivated by acidification to pH 2 with 1 M HCl at 4 °C for 4 days. Samples were then neutralized with 1 M NaOH. Briefly, L-929 cells were seeded into 96-well plates in 0.1 ml DMEM supplemented with 10 % FCS and 1 % l-glutamine and incubated to approximately 90 % confluence. In separate 96-well plates, triplicate 2-fold dilution series of the samples were prepared. Growth medium was removed and replaced with the prepared dilution series and plates were incubated for 24 h. Supernatants were then removed and 0.1 ml DMEM plus 0.5 % FCS containing 300 TCID_50_ (tissue culture infective dose causing 50 % lethality) encephalomyocarditis virus (EMCV) was added. Plates were incubated until complete cytopathic effect was observed in the EMCV controls; this was after approximately 48 h. Infectious medium was replaced with fresh DMEM plus 0.5 % FCS, and 10 μl Cell Proliferation Reagent WST-1 (Roche) per well was added to determine cell viability. The means of the triplicate repeats of the 2-fold dilution series of each sample were plotted and the dilution providing 50 % cell survival was determined. The titre in international units (IU) was determined by reference to a standard curve of an international reference sample (murine IFN-*α*/*β*; GU02-901511, Braton Biotech) run in each assay. Levels of IFN-*α* in mouse sera were determined by capture ELISA (PBL Biomedical Laboratories).

## RESULTS

To determine the role of PKR in SFV-infected cells, primary cultures of MEFs were prepared from C57BL/6×129/Sv/Ev mice (wt) and mice of the same genetic background with a genetic disruption of the PKR gene (PKR^−/−^) (Yang *et al.*, 1995). The PKR genotype of all cultures was confirmed by PCR. Cultures were infected with virus that had been purified through sucrose gradients and washed by centrifugation in order to remove any factors that might be present in virus cell-culture supernatants that could potentially confound these studies.

### PKR does not affect virus RNA replication, but delays virus protein synthesis and production of infectious virus

**Fig. 1. f1:**
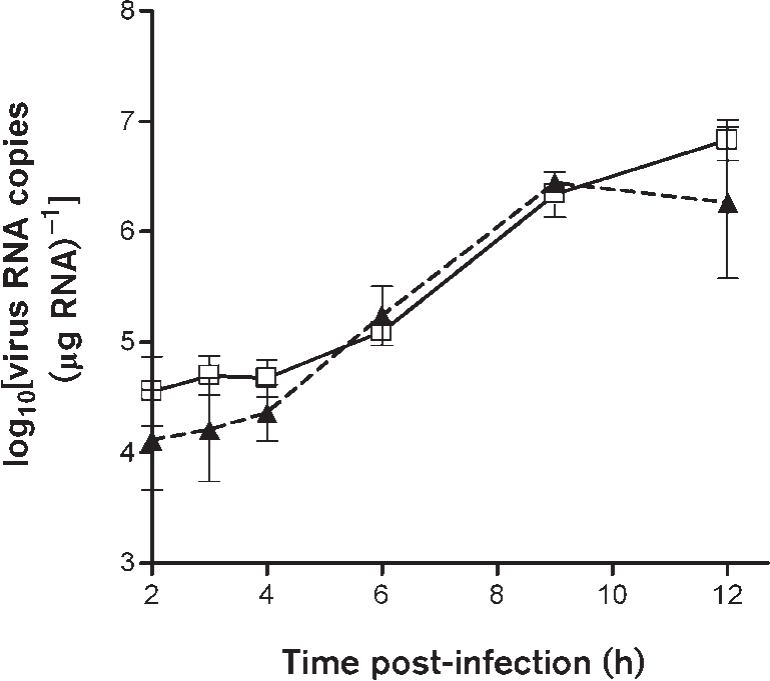
Virus RNA copy number (μg total RNA)^−1^ from wt (□) and PKR^−/−^ (▴) MEFs infected (m.o.i. of 50) with SFV4, as measured by QPCR. Each point is the mean of replicate samples from three parallel cultures. Error bars indicate sd.

**Fig. 2. f2:**
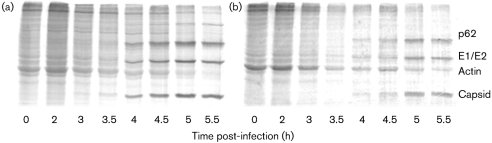
Metabolic labelling of newly synthesized proteins in SFV4-infected (m.o.i. of 50) PKR^−/−^ (a) or wt (b) MEFs. After labelling with [^35^S]Met/Cys, cells were lysed at the indicated time points and equal amounts of protein (μg) were run on 12 % polyacrylamide/SDS gels.

**Fig. 3. f3:**
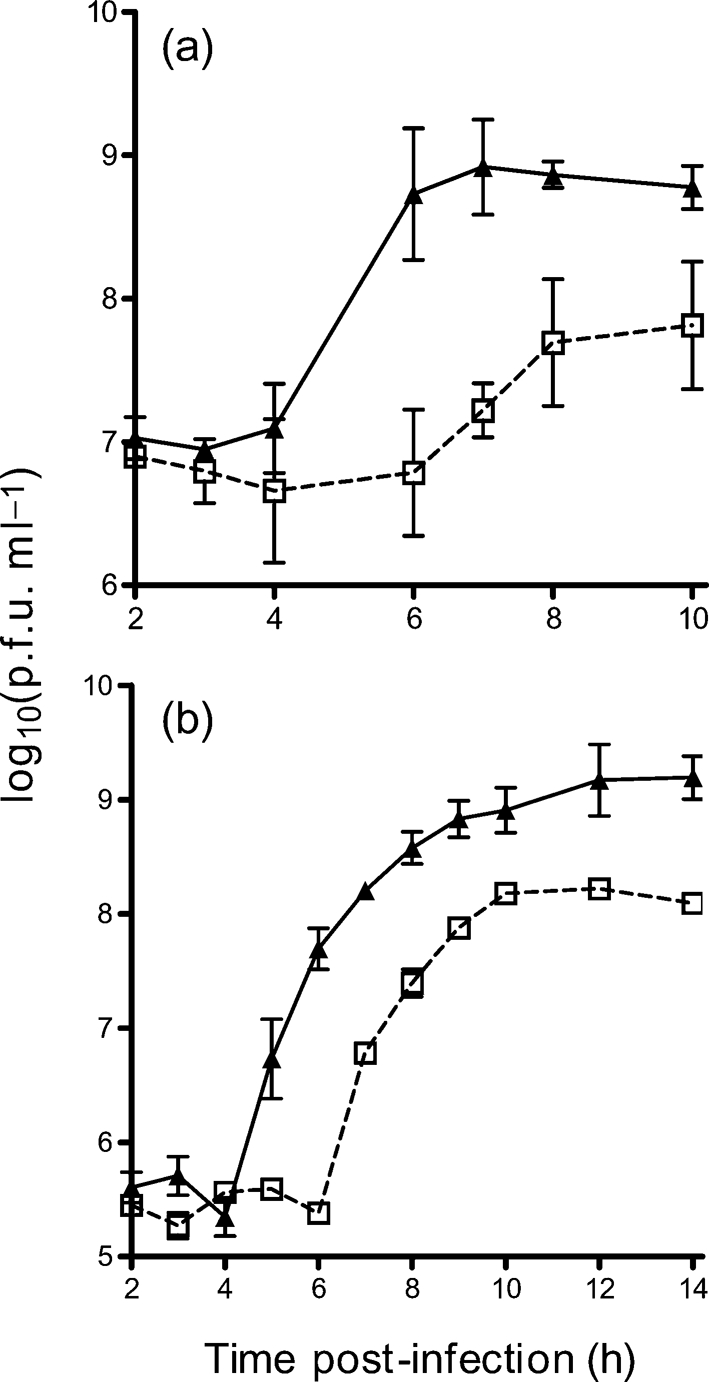
Production of SFV4 virus from wt (□) and PKR^−/−^ (▴) MEFs following infection at an m.o.i. of 50 (a) and an m.o.i. of 1 (b). Each point is the mean of replicate samples from three parallel cultures. Error bars indicate sd.

As measured by immunostaining, SFV4 (m.o.i. of 50) reproducibly infected >96 % of cells in wt and PKR^−/−^ cultures. To determine whether PKR affects early SFV RNA replication, triplicate cultures of wt and PKR^−/−^ MEFs were infected (m.o.i. of 50) with SFV4 and levels of virus RNA wre determined by QPCR. Between 2 and 12 h post-infection, there was no significant difference in the amount of virus RNA in wt and PKR^−/−^ MEFs (Fig. 1). Upon activation, PKR is known to phosphorylate eIF2*α*, leading to reduction or shutoff of host-cell protein synthesis. To compare virus and host-cell protein synthesis, replicate parallel cultures of wt and PKR^−/−^ MEFs were infected with SFV4 and protein synthesis was visualized by labelling with [^35^S]Met/Cys (Fig. 2a, b[Fig f2]). Virus capsid protein was first apparent at 4 h in wt cells, but was apparent 1 h earlier in PKR^−/−^ cells. Between 4 and 5.5 h, levels of all virus structural proteins were higher in PKR^−/−^ cells than in wt cells. A reduction in host-cell protein synthesis was apparent in wt cells from 3.5 h, but was delayed to 5.0–5.5 h in PKR^−/−^ cells. To determine whether earlier virus protein production in the PKR^−/−^ MEFs resulted in earlier production of infectious virus, triplicate cultures of wt and PKR^−/−^ MEFs were infected (m.o.i. of 50 or 1.0) with SFV4 and virus growth curves were determined. Virus production was first apparent at 7 h in wt cells and 2 h sooner in PKR^−/−^ cells (Fig. 3a, b[Fig f3]). In all samples taken after 4 h, virus titres in PKR^−/−^ cultures were consistently and statistically significantly (*t*-test, *P*<0.05) higher than those in wt cultures. It can be concluded that functional PKR had no effect on SFV RNA replication, but delayed virus protein production and release of infectious virus. In the presence of PKR, total virus production was approximately 10-fold lower than in its absence.

### PKR delays cell death

**Fig. 4. f4:**
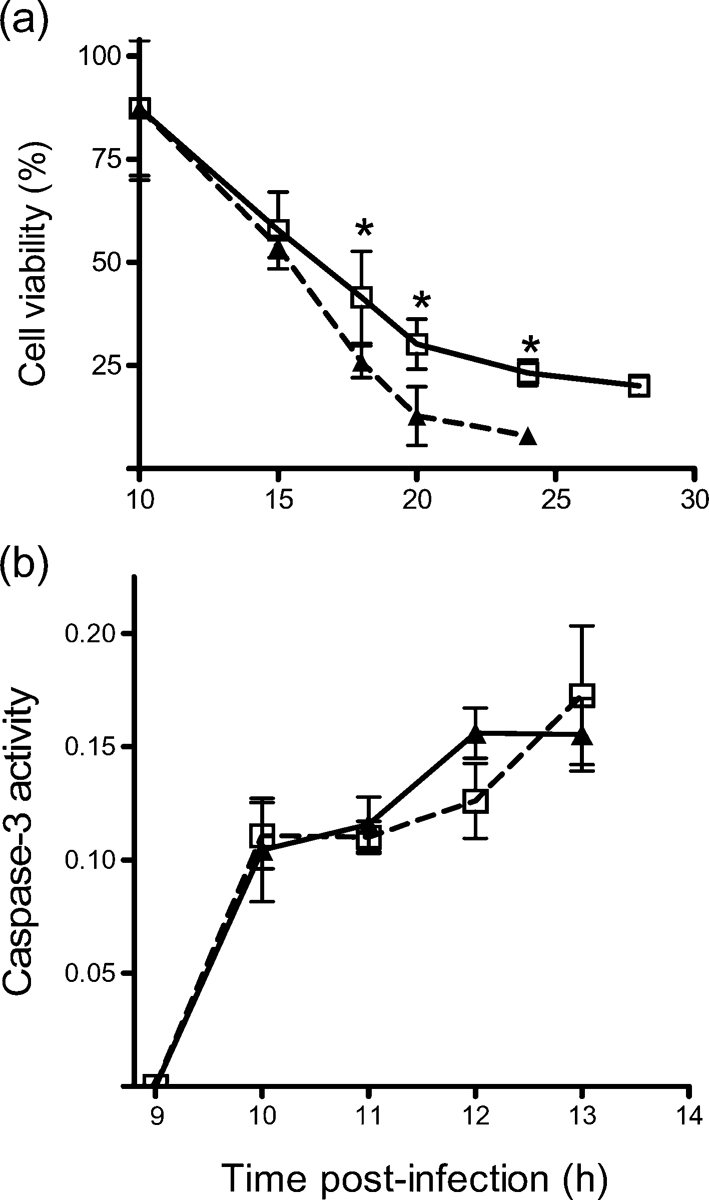
(a) Viability (WST-1) of wt (□) and PKR^−/−^ (▴) MEFs following infection (m.o.i. of 50) with SFV4. Each point represents the mean of four samples. Error bars indicate sd. *P<0.05 (Mann–Whitney test). (b) Levels of activated caspase-3 (arbitrary units), as determined by caspase-3 activation assay. wt (□) and PKR^−/−^ (▴) MEFs were infected with SFV4 (m.o.i. of 50). Cells were lysed and equal amounts (μg) were assayed for caspase-3 activation. Each point represents the mean of three samples. Error bars indicate sd.

Activation of PKR has been reported to initiate apoptosis in response to virus infections and other stimuli (Lee & Esteban, 1994). To determine whether PKR is required for apoptosis in SFV infection, viability of SFV-infected MEFs was determined by WST-1 assay (Fig. 4a[Fig f4]). Virus-infected PKR^−/−^ cultures died more rapidly than virus-infected wt cultures; viability of PKR^−/−^ cultures at 18, 20 and 24 h was significantly lower (Mann–Whitney test, *P*<0.05) than that of wt cultures. In both cell types, caspase-3, a marker of apoptotic cell death, was activated (Fig. 4b[Fig f4]). It can be concluded that, following SFV4 infection, PKR is not required to initiate cell death.

### Verification in NIH-3T3 cells

**Fig. 5. f5:**
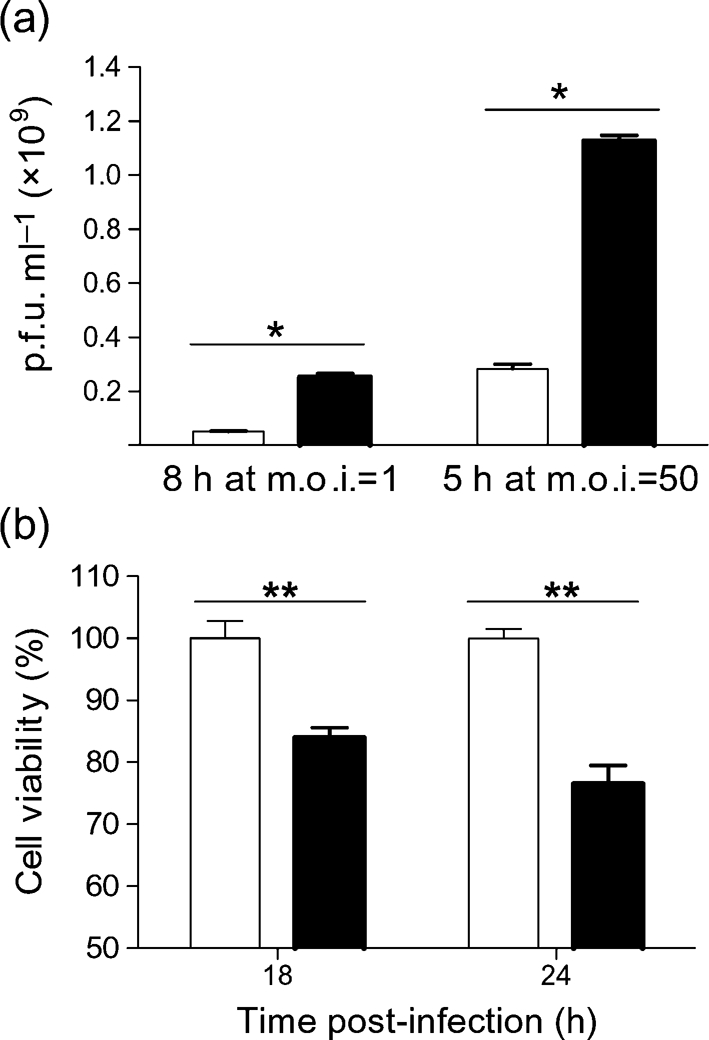
(a) Virus titres in the supernatant and (b) viability of replicate cultures of NIH-3T3 cells pre-treated with control siRNA (empty bars) or PKR siRNA (filled bars) and infected (24 h later) with SFV4 (m.o.i. of 1.0 or 50). Each value is the mean of replicate samples from three parallel cultures. Error bars indicate sd. *P<0.05; **P<0.01 (Mann–Whitney test). The experiment was repeated twice (at each m.o.i.) with similar results.

To determine whether the above findings can be extrapolated to other cell types, levels of PKR were reduced in NIH-3T3 cells by transfection with an siRNA targeting PKR. As determined by Western blotting relative to a control siRNA, the PKR-specific siRNA (at 25, 50 and 100 nM) reduced levels of PKR in NIH-3T3 cells to <50 % by 24 h. This was maintained at 48 h. To determine whether reduced levels of PKR affected virus production and cell viability, parallel cultures were transfected with siRNA (PKR or control at 100 nM) and infected 24 h later with SFV at an m.o.i. of 1.0 or 50. At 5 and 8 h post-infection, supernatants were removed for titration of infectious virus. Cell viability was determined at 18 and 24 h. PKR levels were reduced by 50 % and resulted in increased virus titres (Fig. 5a[Fig f5]) and reduced cell viability (Fig. 5b[Fig f5]). These data support those obtained by using MEFs and verify that PKR acts early in infection to reduce SFV production.

### PKR is not essential for, but contributes to, IFN-*α*/*β* gene expression

**Fig. 6. f6:**
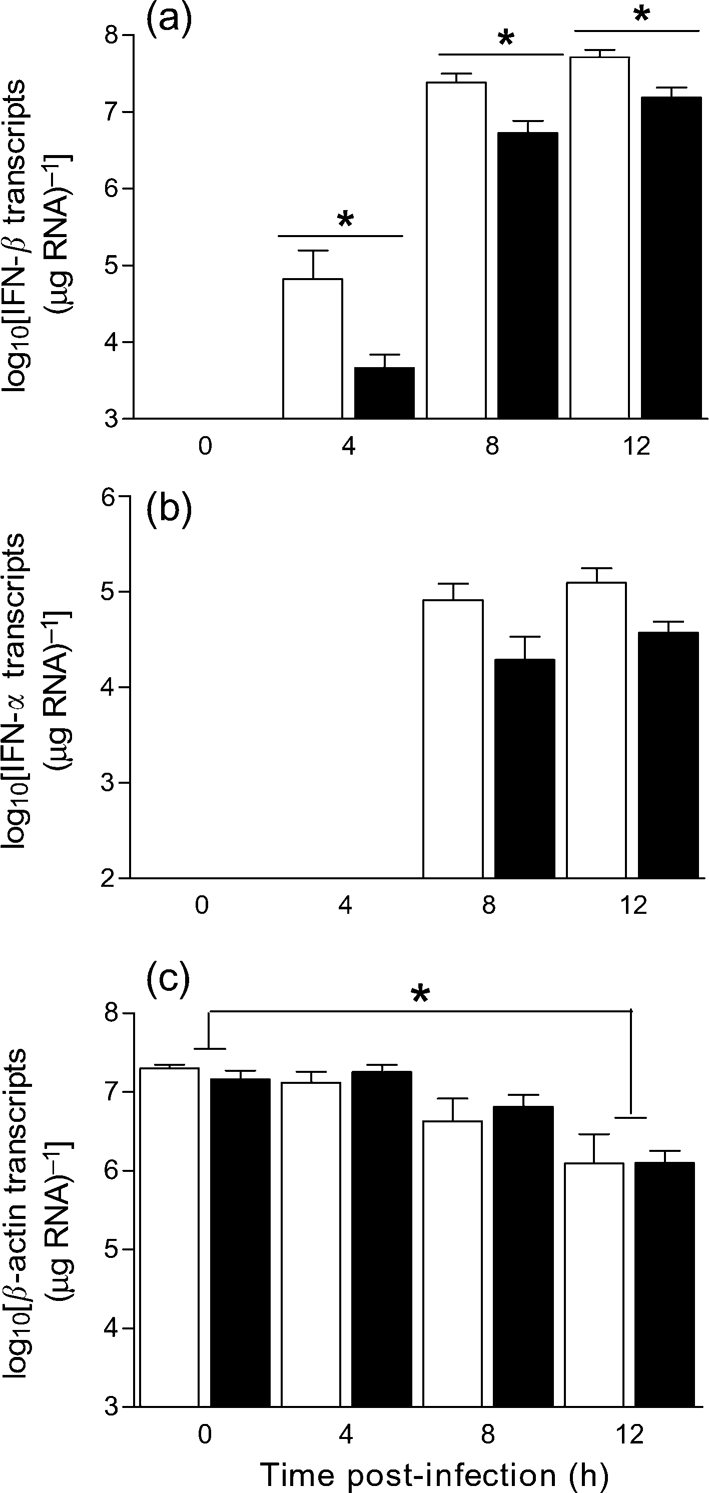
wt (empty bars) and PKR^−/−^ (filled bars) MEFs were grown to confluence and infected (m.o.i. of 10) with SFV4 or mock-infected with PBS. At 0, 2, 3, 4, 8 and 12 h post-infection, cells were harvested and RNA was extracted and reverse-transcribed into cDNA to determine levels of (a) IFN-*β* transcripts, (b) IFN-*α* transcripts and (c) *β*-actin transcripts by QPCR. Each bar represents the mean of two repeat experiments (each done with different preparations of MEFs), each of which included three replicate cultures each assayed in triplicate. The limit of detection is at the intersection of the axes. Error bars indicate sem. *P<0.05 (Mann–Whitney test).

PKR has a role in signal transduction and transcriptional control of gene expression through the I*κ*B/NF-*κ*B pathway (Kumar *et al.*, 1994) and has been suggested in several studies to contribute to activation of the type I IFN response (Garcia *et al.*, 2006; McAllister & Samuel, 2009). To investigate this in SFV infection, levels of IFN gene expression in SFV-infected wt and PKR^−/−^ cells were determined. Levels of IFN-*β* and -*α* transcripts were measured by QPCR. At 2 and 3 h, no IFN-*β* or -*α* transcripts were detectable, and *β*-actin transcript levels were no different from those at the 0 h time point. In both cell types, IFN-*β* transcripts were first detectable at 4 h and demonstrated a dramatic increase (>100-fold) between 4 and 8 h (Fig. 6a[Fig f6]). Absence of PKR did not abrogate IFN-*β* gene transcription; however, at all time points, the mean level of IFN-*β* transcripts in the PKR^−/−^ cells was consistently reduced relative to that in wt cells. These differences were statistically significant (Mann–Whitney test, *P*<0.05) at 4, 8 and 12 h. The ratio of IFN-*β* transcripts produced by wt and PKR^−/−^ cells was 5 : 1 at 8 h and 3 : 1 at 12 h. No IFN-*β* transcripts were detected in mock-infected controls. IFN-*α* transcripts were detectable in both cell types at 8 and 12 h post-infection (Fig. 6b[Fig f6]). Copy numbers were approximately 100-fold lower than those of IFN-*β*. The mean values for the levels of IFN-*α* transcripts expressed by PKR^−/−^ cells were consistently lower than the levels expressed in wt cells, but these differences did not reach statistical significance (Mann–Whitney test, *P*>0.05). No IFN-*α* transcripts were detected in the mock-infected controls.

The reduced level of IFN transcripts in PKR^−/−^ cells indicates that PKR was not required for, but contributed to, IFN gene expression. We reported previously that there are global changes in transcription in SFV-infected MEFs (Breakwell *et al.*, 2007). To determine whether the higher levels of IFN transcripts in wt cells resulted from a specific or global effect of PKR, levels of *β*-actin transcripts were measured in the same samples (Fig. 6c[Fig f6]). With time, the levels of *β*-actin transcripts decreased equally in the two cell types and were significantly different (Mann–Whitney test, *P*<0.05) from those of mock-infected cells at 12 h post-infection (Fig. 6c[Fig f6]); however, there was no difference in *β*-actin transcripts between PKR^−/−^ and wt cells, indicating that the higher levels of IFN transcripts in wt cells resulted from specific enhancement of levels of IFN-*β* transcripts by PKR.

### MEFs deficient in PKR produce less functional IFN than wt MEFs

**Fig. 7. f7:**
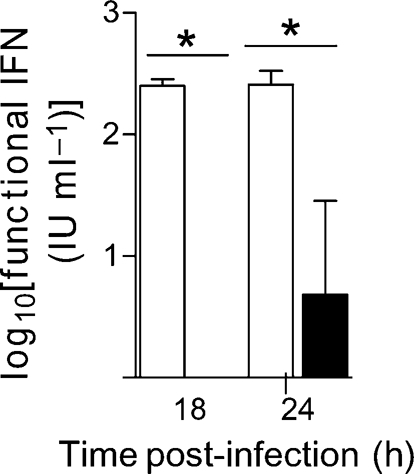
wt (empty bars) and PKR^−/−^ (filled bars) MEFs were grown to confluence and infected (m.o.i. of 10) with SFV4 or mock-infected with PBS. Supernatants sampled from MEFs at 18 and 24 h post-infection were assayed for functional IFN by CPERA. Each bar represents the mean of two repeat experiments (each done with different preparations of MEFs), each of which included four cultures assayed in triplicate. Error bars indicate sem. *P<0.05 (Mann–Whitney test) between wt and PKR^−/−^ cells.

To determine whether levels of functional IFN (translated protein) differ in the presence or absence of PKR, supernatant samples generated from replicate cultures of SFV4-infected or mock-infected wt and PKR^−/−^ MEFs were assayed by cytopathic effect reduction assay (CPERA) for levels of functional IFN (Fig. 7). No IFN was detected in mock-infected controls. The level of functional IFN in infected PKR^−/−^ cells was reduced significantly (Mann–Whitney test, *P*<0.05) relative to that in infected wt cells (Fig. 7). The ratio of functional IFN produced by wt and PKR^−/−^ cells was >250 : 1 at 18 h and 50 : 1 at 24 h.

### PKR is not required to protect adult mice, but contributes to the rate of virus clearance from the brain

**Fig. 8. f8:**
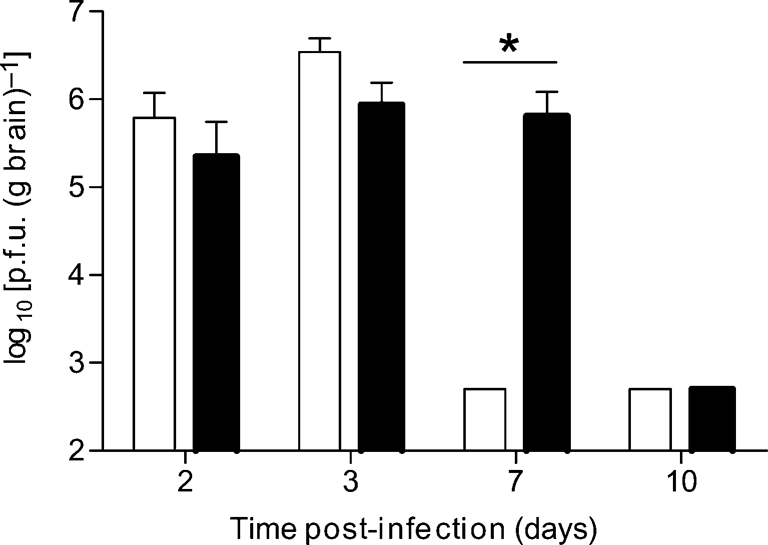
Groups (*n*=25) of 4–5-week-old wt and PKR^−/−^ mice were infected ip with 5000 p.f.u. SFV A7(74). At various times post-infection, brain virus titres were determined by plaque assay. Each bar represents the mean (*n*=6) virus titre from wt (empty bars) or PKR^−/−^ (filled bars) mouse brains. Error bars indicate sd. The limit of detection of the assay was 10^2.4^ p.f.u. (g brain)^−1^. *P<0.05 (Mann–Whitney test).

We have shown previously that an intact type I IFN response is required to protect adult mice from SFV infection (Fragkoudis *et al.*, 2007). To determine whether PKR is required for this protection, groups (*n*=12) of 4–5-week-old wt and PKR^−/−^ mice were infected ip with 5000 p.f.u. SFV4 or SFV A7(74) and their survival was monitored for 14 days. The PKR genotype of all mice was determined by PCR. Both wt and PKR^−/−^ mice survived the infection, demonstrating that PKR was not required to protect adult mice from SFV infection. To determine whether PKR has an effect on the course of infection, groups of 4–5-week-old wt and PKR^−/−^ mice were infected ip with neuroinvasive SFV A7(74) and serum IFN titres and brain virus titres were determined at various times post-infection. Absence of PKR resulted in lower titres of serum IFN. The mean serum titre (*n*=6) of IFN at day 2 in wt and PKR^−/−^ mice was 412 and 49 pg ml^−1^, respectively (Mann–Whitney test, *P*<0.01). Titres of infectious brain virus were not significantly different at days 2 and 3, but whilst infectious virus had been cleared from the brains of wt mice by day 7, titres of infectious virus remained high in PKR^−/−^ mice at this time (Fig. 8).

## DISCUSSION

In SFV-infected MEFs, the presence of PKR delayed virus protein synthesis, infectious virus production and apoptotic cell death. PKR was not required for apoptosis, induction of type I IFN gene transcription or production of functional IFN, but PKR greatly augmented the IFN response. In mice, PKR was not necessary for type I IFN-mediated protection from SFV infection (Fragkoudis *et al.*, 2007), but contributed to levels of serum IFN and clearance of infectious virus from the brain.

It has been reported that cells from these PKR^−/−^ mice (Yang *et al.*, 1995) express a truncated PKR that cannot bind dsRNA, but remains catalytically active (Baltzis *et al.*, 2002); if so, our results may be attributed to deletion of the N-terminal domain of PKR. However, the presence of a truncated PKR in these mice has not been verified and these cells generally continue to be considered PKR-null.

Between 2 and 12 h post-infection, there was no significant difference in the amounts of virus genomic RNA produced in wt and PKR^−/−^ MEFs. However, in the presence of PKR, virus structural proteins were produced more slowly. PKR also delayed the release of infectious virus. The delay, approximately 2 h, was a considerable difference given the short replication time of this virus. The presence of PKR resulted in a 90 % reduction in virus yield from the infected culture. This would have a much larger cumulative effect on multiple rounds of virus infection in tissues, providing a powerful antiviral mechanism. A similar enhancement of infectious virus production has been observed in SV-infected PKR^−/−^ mouse bone marrow-derived dendritic cells (Ryman *et al.*, 2002). Taken together, these two studies show that PKR exerts a powerful antiviral effect early in alphavirus infection.

PKR was not required to shut off host-cell protein synthesis, but sped up this response. PKR is one of four known cellular kinases that can phosphorylate eIF2*α* (de Haro *et al.*, 1996). The shutoff of host-cell protein synthesis in the absence of PKR could have resulted from the activity of other eIF2*α* kinases, such as general control non-derepressible-2 (GCN2) or PKR-like endoplasmic reticulum kinase (PERK). PERK is activated by the presence of unfolded proteins in the endoplasmic reticulum, a cell stress response activated following infection with other enveloped RNA viruses (Medigeshi *et al.*, 2007). Induction of host-cell protein shutoff by PERK would be expected to occur later than that following activation of PKR by dsRNA, and PERK-mediated shutoff would be consistent with the delay in shutoff observed in PKR^−/−^ cells. In SV-infected cells in culture, PKR also has a role in translational shutoff, but again it is not the only mechanism involved (Gorchakov *et al.*, 2004; Ryman *et al.*, 2005). GCN2 is also activated in SV-infected cells and blocks early virus translation of genomic RNA (Berlanga *et al.*, 2006).

The mechanism by which production of virus structural protein and release of infectious virus are delayed by PKR is not clear, but three possibilities should be considered. Firstly, that PKR affects virus replication by shutting off cap-mediated eIF2*α*-dependent host-cell and virus replicase protein synthesis. The virus subgenomic translational enhancer allows translation of the subgenomic RNA after eIF2*α* phosphorylation, but translation of structural proteins could nevertheless be more efficient in the absence of host-cell shutoff. Secondly, that PKR affects virus replication via the IFN response. Virus structural proteins were first detected in PKR^−/−^ cells at 3 h and in wt cells at 4 h, whilst IFN-*β* transcripts were first detectable between 3 and 4 h and were more abundant in wt cells than in PKR^−/−^cells. It is possible but, given the timing, unlikely that autocrine effects of IFN unrelated to PKR delayed virus protein synthesis and release of infectious virus. Recently, an IFN-activated system that inhibits cap-dependent translation of SV, but not cellular, RNA has been demonstrated (Tesfay *et al.*, 2008). Thirdly, that PKR affects virus replication by activating other, non-IFN-inducible antiviral mechanisms, perhaps autophagy (Orvedahl & Levine, 2008).

PKR was not required for apoptotic cell death or IFN gene expression following SFV infection. This is consistent with results observed for SV infection in bone marrow-derived dendritic cells (Ryman *et al.*, 2002). Although not required, the presence of PKR increased the levels of both IFN-*β* and IFN-*α* transcripts. Given that IFN-*α* expression is augmented by IRF-7, a transcription factor induced by IFN-*β* (Marie *et al.*, 1998), the higher levels of IFN-*α* are likely to have resulted from the higher levels of IFN-*β*. Consistent with this, IFN-*β* transcripts were first detected at 4 h and IFN-*α* transcripts at 8 h, and levels of IFN-*α* transcripts were always proportional to those of IFN-*β*. The differences in IFN transcripts between cells with and without PKR did not result from global differences in cellular transcription or degradation, as reductions over time in levels of *β*-actin transcripts were the same in wt and PKR^−/−^ cells. This temporal reduction in *β*-actin gene expression is consistent with our previous finding that SFV infection suppresses global cellular gene transcription (Breakwell *et al.*, 2007), a phenomenon also observed with SV (Gorchakov *et al.*, 2005).

The mechanism by which IFN-*β* gene expression is initiated in alphavirus-infected cells remains to be determined. In addition to PKR, other sensors of infection include RIG-I and mda-5, which respond to virus RNA and initiate IFN expression following infection by other RNA viruses (Andrejeva *et al.*, 2004; Gitlin *et al.*, 2006; Kato *et al.*, 2006). In SFV-infected MEFs, IFN responses were considerably higher in the presence of PKR. The difference was greater for functional IFN (>250 : 1 at 18 h and 50 : 1 at 24 h) than for IFN gene transcripts (8 : 1 at 8 h and 3 : 1 at 12 h). The higher levels of IFN transcripts presumably result from more efficient activation of IFN gene expression in the presence of signals derived from PKR activation. These higher levels of IFN transcripts could directly explain the higher levels of functional IFN; however, levels of functional IFN were increased more profoundly than levels of IFN transcripts, and other factors, perhaps acting at the translational level, are also likely to be important.

A type I IFN response is required to protect mice from SFV4 infection (Fragkoudis *et al.*, 2007). The fact that PKR was not required for this protection is consistent with the finding that it is not required for an IFN response in MEFs. However, the *in vivo* situation is more complex than that *in vitro*, as many different cell types contribute to IFN production and these may utilize different mechanisms of IFN induction. In the absence of PKR, levels of IFN in serum were reduced significantly. These reduced IFN levels are probable causes of the slower clearance of infectious virus from the brains of PKR^−/−^ mice, either directly or perhaps indirectly via their effect on adaptive immune responses responsible for SFV clearance from the brain (Amor *et al.*, 1996). However, PKR is also important for the initiation of autophagy and this may be important in protecting the mature post-mitotic neurons of the CNS (Orvedahl & Levine, 2008).
